# Mutual distrust: understanding and bridging the science to policy gap

**DOI:** 10.1186/1748-5908-10-S1-A48

**Published:** 2015-08-20

**Authors:** Sarah E Gollust, Jane W Seymour, Maximilian J Pany, Adeline Goss, Zachary Meisel, David Grande

**Affiliations:** 1 Division of Health Policy & Management, School of Public Health, University of Minnesota, Minneapolis, MN 55455, USA; 2 Division of General Internal Medicine, Perelman School of Medicine, University of Pennsylvania, Philadelphia, PA 19104, USA; 3 Swarthmore College, Swarthmore, PA 19081, USA; 4 Perelman School of Medicine, University of Pennsylvania, Philadelphia, PA 19104, USA; 5 Department of Emergency Medicine, Perelman School of Medicine, University of Pennsylvania, Philadelphia, PA 19103, USA

## Background

The production of policy-relevant research is necessary, but not sufficient, to promote its utilization in policy. Decision-makers must believe it has value, and researchers need the desire and skills to translate their work effectively. Our objective was to understand the perspectives of state policymakers and health researchers on the barriers and facilitators to translation of health evidence into the policy process, with an emphasis on the potential of social media.

## Methods

We conducted interviews with 215 health services and health policy researchers at the 2013 Academy Health Research meeting and 40 staffers and legislators at the 2013 National Conference of State Legislatures meeting. Transcripts were analyzed qualitatively through an inductive process, comparing emergent themes across the two groups.

## Results

Researchers and policymakers were mutually distrustful: many policymakers questioned researchers' credibility and their agendas, arguing that science can be too easily "spun" to rely on it in making decisions. Similarly, researchers expressed cynicism about policymakers' interest in and ability of using evidence to make decisions, suggesting that money and politics reigned. Despite these attitudinal barriers, some policymakers and researchers wanted to reach the other more effectively. However, researchers felt unprepared to undertake what they believed was required for policy impact. Policymakers described the paramount importance of credible information sources with whom they have established relationships. Both groups were concerned about the time it takes to communicate or incorporate research evidence. A plurality of neither group expressed confidence that social media, at present, could overcome these gaps.

## Conclusions

This research identifies future dissemination and implementation research imperatives: to develop and test efficient dissemination interventions that are viewed as highly credible by both researchers and policymakers and can increase personal contact, and ultimately trust, between groups.

